# 

**Published:** 1987-08

**Authors:** 


W1 BR323

NO.3 1987

--------SEQ: B33740000

RISTOL MEDICO-CHIRURGICAL

OURNAL

### BRISTOL MEDICO-CHIRURGICAL JOURNAL

VOLUME 102(iii) AUGUST 1987

NATIONAL LIBRARY
OCT 09 1987
OF MEDICINE

The AIDS Epidemic and its control.
The Origin of AIDS
Adverse Reaction to Sulphasalazine.
Axillary Aneurysm.
Ten years on
News and comment from at home and abroad.
Etc. Etc.

IN ASSOCIATION WITH

BRISTOL MEDICINE

THE MEDICAL JOURNAL OF THE SOUTH WEST

